# Duodenum inversum presenting with intestinal obstruction in a 40-day-old infant: a case report

**DOI:** 10.3389/fmed.2026.1923796

**Published:** 2026-09-21

**Authors:** Elisa Pani, Federica Varner, Fabio Beretta, Silvia Bisoffi, Federica Fati, Giosuè Mazzero, Clara Revetria, Hamid Reza Sadri, Daniela Codrich

**Affiliations:** 1 Department of Pediatric Surgery, Hospital Santa Chiara ASUIT, Trento, Italy; 2 Pediatric Surgery, Woman and Child Health Department, Hospital of Padua, Padua, Italy

**Keywords:** congenital duodenal anomaly, duodenum inversum, infant, pediatric intestinal obstruction, recurrent postprandial vomiting, upper gastrointestinal contrast study

## Abstract

**Background:**

Duodenum inversum is a rare congenital anomaly characterized by an abnormal course of the duodenum with preservation of a normally positioned duodenojejunal junction. Clinical presentation is highly variable, ranging from incidental findings to symptoms of upper gastrointestinal obstruction. Due to its rarity, diagnosis and management remain challenging, particularly in pediatric patients.

**Case presentation:**

We report the case of a 40-day-old female infant presenting with recurrent postprandial vomiting and poor weight gain since the first days of life. Initial clinical, metabolic, and ultrasonographic evaluations were unremarkable, including exclusion of hypertrophic pyloric stenosis. A trial of conservative management provided only temporary improvement. A subsequent upper gastrointestinal contrast study suggested an abnormal duodenal configuration, while endoscopic and radiological reassessment demonstrated a reversed and tortuous duodenal course consistent with duodenum inversum. Following recurrence of symptoms and failure of conservative measures, the patient underwent exploratory laparotomy, which revealed a mobile duodenum with fibrotic adhesions between the duodenum, colon, and retroperitoneum causing functional obstruction. Adhesiolysis restored normal duodenal anatomy. The postoperative course was uneventful, with complete resolution of symptoms and appropriate growth at 6-month follow-up.

**Conclusion:**

Although duodenum inversum is typically asymptomatic, it may rarely present in infancy with significant obstructive symptoms. Fibrotic adhesions are thought to contribute to the pathophysiology of obstruction in symptomatic cases, although their precise role remains incompletely understood. Upper gastrointestinal contrast studies remain the main diagnostic tool, while surgical treatment is reserved for patients with persistent or complicated obstruction. Awareness of this entity is essential to avoid misdiagnosis and to ensure appropriate management. To our knowledge, our case represents the youngest patient requiring surgery reported in the literature.

## Introduction

Duodenum inversum is an uncommon congenital anomaly characterized by an atypical course of the duodenum. In affected individuals, the third portion of the duodenum ascends posteriorly before crossing the midline above the level of the pancreas (1, 2). In contrast to other intestinal rotational abnormalities, the duodenojejunal junction retains its normal anatomical position.

The underlying pathogenesis of this condition has not yet been fully elucidated. One proposed mechanism is the persistence of the dorsal mesentery, resulting in excessive mobility of the duodenum (3). Since its first description, about 20 cases have been reported in the medical literature, most involving adult patients, with 10 cases described in the pediatric population (Table 1), and a variety of therapeutic strategies have been described (1–15). Clinical manifestations vary considerably, ranging from incidental findings in asymptomatic individuals to severe presentations requiring surgical intervention.

**Table 1:** Previously reported cases of pediatric duodenum inversum.

Report	Year	Patient age	Gender	Presenting symptoms	Diagnostic imaging	Treatment
Long et al. (9)	1999	16 y/o	Female	Nausea, vomiting, cramping	UGI	Exploratory laparotomy
Kim et al. (3)	2013	1 m/o	Male	GERD	UGI	Diagnostic laparoscopy
Patel et al. (7)	2016	2 m/o	Male	Fussiness, stiffening	UGI	Nissen's fundoplication
Patel et al. (7)	2016	2 m/o	Male	Stiffening, gasping, spitting up		Proton pump inhibitors
Dogan et al. (2)	2016	12 y/o	Female	Nausea, vomiting	UGI	Proton pump inhibitors
Menchise et al. (8)	2016	10 y/o	Female	Abdominal pain, bilious emesis	abdominal US, abdominal X-ray, UGI	EGD, nasojejunal tube
Winborne and Sims (16)	2023	7 m/o	Female	Nonbilious emesis	UGI	Famotidine
Sharma et al. (17)	2023	16 y/o	Male	Nonbilious emesis	abdominal CT, UGI, abdominal X-ray	Amitriptyline, cyproheptadine, and total parenteral nutrition
Srivatsa et al. (18)	2024	13 y/o	Male	Bilious emesis	abdominal X-ray, UGI, abdominal US	Surgical correction
Ahmadian et al. (14)	2025	6 y/o	Male	weight loss, nausea, vomiting, abdominal pain,	UGI, EGD	cyproheptadine

UGI, fluoroscopy upper gastrointestinal exam series; abdominal US, abdominal ultrasound; EGD, esophagogastroduodenoscopy; abdominal CT, abdominal computed tomography scan.

GERD, gastroesophageal reflux disease.

Awareness of this rare anatomical variation is important, as it may be mistaken for intestinal malrotation, potentially resulting in unnecessary surgical treatment. Although many individuals with duodenum inversum remain asymptomatic, the anomaly has been associated with several clinical conditions, including duodenitis, proximal duodenal obstruction, and pancreatitis (4–11). Due to its rarity, both diagnosis and management remain challenging for clinicians.

Herein, we describe the case of a 40-day-old female infant with symptomatic duodenum inversum who ultimately required surgical correction. To our knowledge, our case represents one of the youngest patient requiring surgery reported in the literature.

## Case report

We report the case of a 40-day-old female infant who was initially admitted to the pediatric ward and subsequently transferred to the pediatric surgery unit for recurrent vomiting since the first days of life. She was the second child, born at 39 weeks + 1 day of gestation. The pregnancy was uneventful, and delivery was spontaneous and uncomplicated. At birth, she adapted well to extrauterine life, with Apgar scores of 9 and 10 at 1 and 5 min, respectively. Her birth weight was 2710 g.

Upon admission, she was in good general condition, and physical examination revealed no abnormalities.

Two abdominal ultrasound examinations were performed, both of which showed no pathological findings, specifically excluding hypertrophic pyloric stenosis. Venous blood gas analysis and plasma ammonia levels were within normal limits, eliminating the need for further metabolic investigations. Additionally, electrocardiography (ECG), cranial ultrasonography, and neurological evaluation were unremarkable.

A trial of feeding with hydrolyzed formula combined with positional maneuvers was initiated, starting with 30 mL per feed and gradually increasing to 60 mL. However, vomiting persisted with increasing feeding volumes. A nasogastric tube was then placed, resulting in significant clinical improvement; feeds administered via the tube were well tolerated, even at age-appropriate volumes. When oral feeding was resumed, vomiting recurred, prompting an upper gastrointestinal (UGI) contrast study. The examination showed no overt pathological findings, with normal passage of contrast through the esophagus and pylorus, although the position of the duodenal C-loop could not be clearly determined (Figure 1).

**Figure 1:**
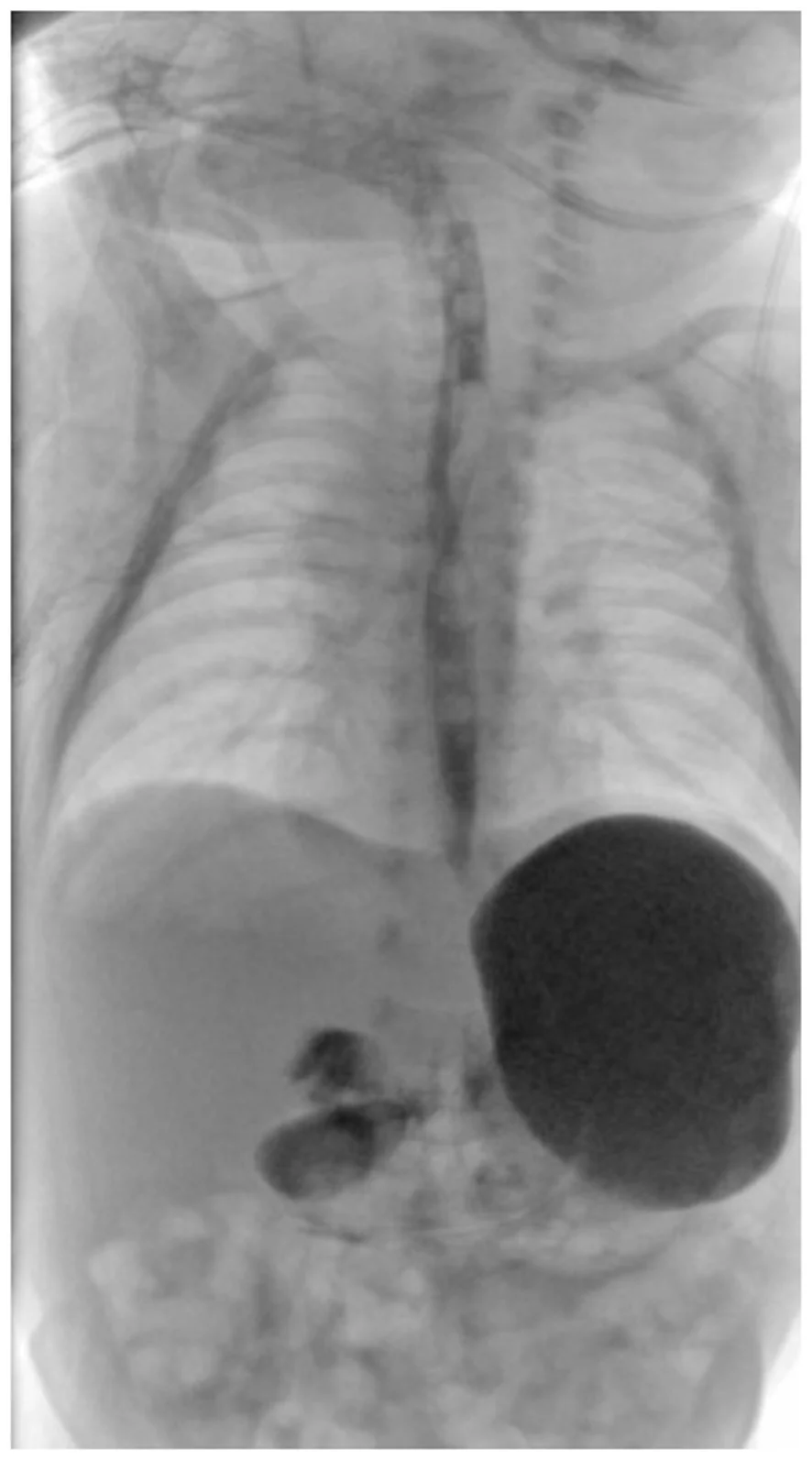
Contrast study of the upper gastrointestinal tract demonstrating an abnormal duodenal course.

Over the following days, the infant resumed oral feeding with progressively increasing volumes, without further episodes of vomiting, and demonstrated satisfactory weight gain. She was discharged with weekly outpatient follow-up.

Approximately 4 weeks later, she presented to the emergency department with recurrent postprandial vomiting and weight loss. Consequently, esophagogastroduodenoscopy (EGD) was performed to exclude incomplete intraluminal diaphragms, together with a repeat UGI contrast study under endoscopic guidance to evaluate the proximal duodenum for possible intestinal fixation abnormalities, particularly malrotation or duodenum inversum.

EGD revealed no significant abnormalities except for a posteriorly displaced pyloric antrum, which prevented clear visualization of the pyloric orifice. The UGI study, performed by injecting contrast into the pyloric antrum, demonstrated a reversed, hook-shaped course of the duodenum (Figure 2). A transpyloric nasogastric tube was then placed under fluoroscopic guidance. An abdominal CT scan was also performed to rule out vascular anomalies (e.g., Wilkie's syndrome); it confirmed the abnormal orientation of the pyloric region and proximal duodenum.

**Figure 2:**
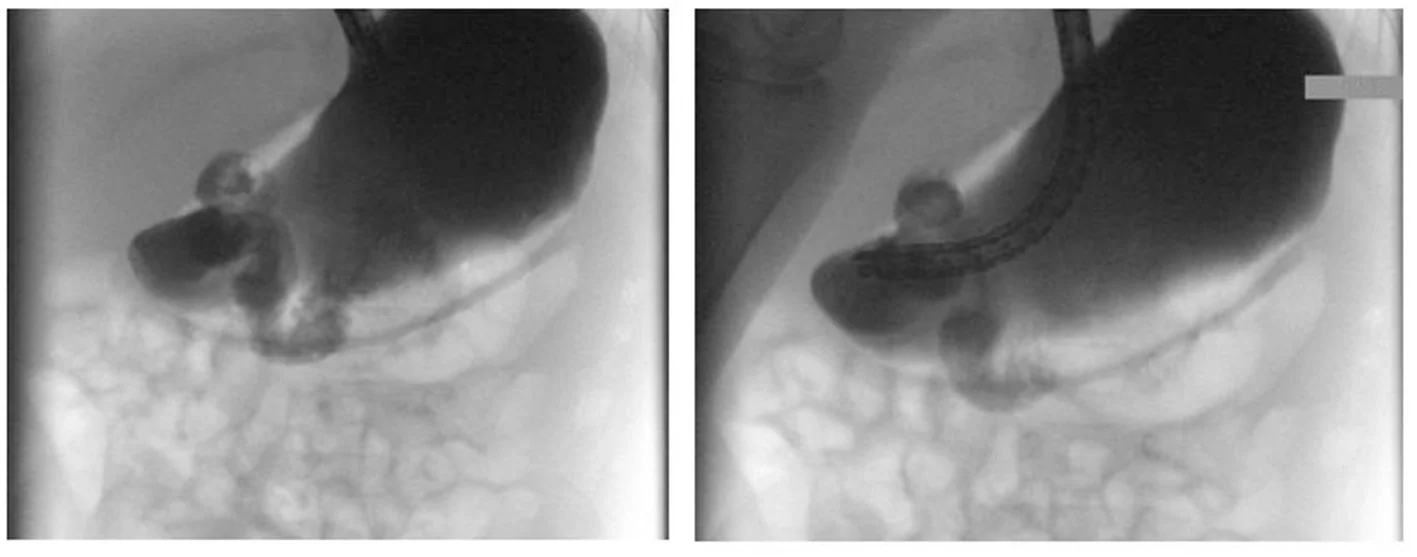
Contrast-enhanced reassessment during endoscopy demonstrating a reversed and hooked course of the duodenum.

The differential diagnosis in this case included severe gastroesophageal reflux, vascular anomalies such as superior mesenteric artery syndrome, annular pancreas, intraluminal diaphragms, pyloric anomalies, and duodenum inversum. We decided to perform an exploratory laparotomy to confirm our diagnostic hypothesis, as enteral feeding had become impossible because the infant developed persistent vomiting and weight loss.

The laparotomy revealed a mobile duodenum with the first portion stretched anteriorly by adhesions to the colon. The obstructive band was a thick, fibrinous tissue. No annular pancreas was identified. Adhesions between the first portion of the duodenum and the colon, as well as between the second portion of the duodenum and the retroperitoneum, were released. No intraluminal diaphragms were identified. Following mobilization of the first and second portions of the duodenum, the normal course of the duodenal C-loop was restored, with the pancreas in its normal medial position. The ligament of Treitz was in its orthotopic position, and the cecum and appendix were normally located. A nasojejunal tube was placed at the end of the procedure.

The postoperative course was uneventful. The patient initially received parenteral nutrition and enteral feeding via the nasojejunal tube. Oral feeding was resumed on postoperative day 3 and was well tolerated, with gradually increasing feeding volumes. The nasojejunal tube was removed on postoperative day 9. She was discharged on postoperative day 14 in good general condition, with satisfactory weight gain and exclusive oral feeding.

At the 6-month follow-up, no complications had occurred. The infant was growing well, remained in good general condition, had successfully started complementary feeding, and had experienced no further episodes of vomiting.

## Discussion

Duodenum inversum is a rare congenital anatomical variant characterized by an atypical course of the duodenum. It may present with a broad spectrum of clinical manifestations and is often identified incidentally. In pediatric patients, reported symptoms include gastroesophageal reflux disease (GERD), abdominal pain, nausea, vomiting, and irritability (Table 1). The incidence of duodenum inversum in the general population remains unknown. Since its first description in the medical literature in 1950, fewer than 20 confirmed cases have been reported (1–15).

Reported cases have demonstrated a wide range of clinical presentations, from asymptomatic individuals diagnosed postmortem to patients with gastrointestinal (GI) obstruction, gastroesophageal reflux, duodenitis, acute pancreatitis, and peptic ulcer disease. Despite these clinical observations, the precise etiology and pathogenesis of duodenum inversum remain unclear.

A marked male predominance has been reported, with a male-to-female ratio of approximately 4:1 (3, 4, 10). The principal imaging modality for diagnosing duodenum inversum is the fluoroscopic upper gastrointestinal (UGI) contrast study. Characteristic radiological findings include an abnormal course of the duodenum, reflux of contrast from the second to the first portion of the duodenum, and delayed contrast transit with duodenal stasis (1, 4).

Radiological subtypes of this condition were first described by Feldman and Morrison in 1950, who classified duodenum inversum into four subtypes: Type 1, characterized by complete duodenal inversion with absence of the duodenal curve; Type 2, characterized by preservation of the duodenal curve; Type 3, characterized by a redundant duodenal curve; and Type 4, defined as duodenum inversum associated with intestinal malrotation. Despite this detailed classification, its clinical utility has not been established, mainly because of the rarity of the condition (3).

Nevertheless, clinicians should consider this anatomical variant in the differential diagnosis of chronic GERD or acute GI obstruction in both pediatric and adult patients.

Most patients with duodenum inversum remain asymptomatic throughout life. Conservative management is the treatment of choice in the absence of associated obstruction and typically includes acid-suppressive therapy and antispasmodic agents. Rare case reports have described patients presenting with symptoms secondary to proximal bowel obstruction (3, 9). Fibrotic adhesions are thought to contribute to the pathophysiology of obstruction in symptomatic cases (13), as observed in our patient, and requires surgical release, although their precise role remains incompletely understood. More extensive procedures aimed at “straightening” the duodenum are generally not recommended.

In our patient, intestinal malrotation had already been excluded by the fluoroscopic UGI contrast study, and the normal position of the ligament of Treitz was confirmed during exploratory laparotomy. However, the course of the duodenum was abnormal. Fibrous bands were identified between the first portion of the duodenum and the colon, as well as between the second portion of the duodenum and the retroperitoneum, likely resulting in duodenal obstruction, as previously described.

When obstructive symptoms are clinically evident, as in our patient, surgical treatment is the preferred management strategy. To date, no specific operative approach has been shown to be superior for the different anatomical variants of duodenum inversum. Surgical treatment is generally directed toward relieving the mechanical obstruction responsible for the patient's symptoms. In previously reported cases, the most common intraoperative finding has been dense fibrous adhesions, which are thought to play a possible role in the development of partial duodenal obstruction. A similar finding was observed in our patient during surgical exploration (3, 9, 13).

The present report represents, to our knowledge, one of the youngest patient requiring surgery reported in the literature. An UGI contrast study remains the primary diagnostic modality for identifying this anatomical anomaly and distinguishing it from other more common causes of upper gastrointestinal obstruction, particularly intestinal malrotation.; however, we believe that EGD may also be useful to exclude pyloric anomalies and/or intraluminal diaphragms and to aid surgical planning when operative management is required.

Conservative treatment with anti-reflux medication may be appropriate for patients with mild symptoms; however, patients with intestinal obstruction or those who fail to respond to medical therapy generally require surgical intervention, often beginning with diagnostic laparoscopy or laparotomy.

## Conclusion

Although duodenum inversum is most often clinically silent, it may occasionally present in early infancy with clinically significant symptoms, particularly those related to upper gastrointestinal obstruction and feeding intolerance. Fibrous adhesions appear to play a possible role in the pathophysiology of obstruction in symptomatic patients, as they may distort the normal duodenal anatomy and cause intermittent or persistent luminal narrowing.

We describe the clinical course and surgical management of an infant with symptomatic duodenum inversum because this case highlights the importance of recognizing this rare anomaly to guide appropriate diagnostic evaluation and timely medical or surgical management.

## Data Availability

The original contributions presented in the study are included in the article/supplementary material, further inquiries can be directed to the corresponding author.
